# Complete reference genomes of *Haemophilus ducreyi* isolates ITG3105, CIP76118, and TMA3542, using Oxford Nanopore and Illumina sequencing

**DOI:** 10.1128/mra.01425-25

**Published:** 2026-05-18

**Authors:** Daniel Golparian, Daniel Schröder, Magnus Unemo

**Affiliations:** 1 World Health Organization Collaborating Centre for Gonorrhoea and other Sexually Transmitted Infections, Department of Laboratory Medicine, Microbiology, Faculty of Medicine and Health, Örebro University 6233, Örebro, Sweden; 2 Institute for Global Health, University College London (UCL) 4919, London, United Kingdom; Nanchang University, Nanchang, Jiangxi, China

**Keywords:** *Haemophilus ducreyi*, chancroid, complete reference genomes

## Abstract

We report the complete reference genome sequences of three *Haemophilus ducreyi* isolates (ITG3105, CIP76118, and TMA3542), generated using Oxford Nanopore and Illumina sequencing, and hybrid assemblies. Each genome has a closed circular chromosome; ITG3105 and TMA3542 also have plasmids. These reference genomes support genomic, epidemiological, and antimicrobial resistance surveillance of *H. ducreyi*.

## ANNOUNCEMENT

*Haemophilus ducreyi* causes chancroid, which has mainly disappeared as a sexually transmitted infection in many countries where it was previously endemic (1–4). However, in the recent decade, *H. ducreyi* was also shown to cause cutaneous ulceration in children, especially in yaws-endemic regions (3, 4). The global epidemiology of *H. ducreyi* infections remains unknown because syndromic management is mostly used, and quality-assured, sensitive diagnostic assays are lacking, especially in resource-limited settings (1–6). Due to the recently described high prevalence in, for example, Malawi (7), and the rising importance of genomic and antimicrobial resistance (AMR) surveillance for sexually transmitted and skin pathogens, complete reference genomes are needed for *H. ducreyi*. We report high-quality, complete reference genomes for three *H*. *ducreyi* isolates (ITG3105, CIP76118, and TMA3542) that will support genomic, epidemiological, and AMR surveillance.

The *H. ducreyi* isolates (Table 1) were grown on chocolate agar supplemented with IsoVitaleX (BD Diagnostics) at 33°C ± 1°C in a 5% CO₂-enriched atmosphere for 48–72 h, and species verified using MALDI-TOF MS (Bruker). Genomic DNA was extracted using the Nanobind HMW DNA extraction kit (PacBio). Illumina DNA Library Prep (Illumina) and Ligation Sequencing Kit V14 SQK-LSK114 (Oxford Nanopore Technologies [ONTs]), without DNA shearing or size selection, were used for short- and long-read library preparations, respectively. Sequencing was performed on a NextSeq 550 platform (2 × 150 bp reads) and an Mk1D with an R10.4 flow cell (ONT). DNA and libraries were quality controlled using Qubit (Thermo Fisher) and Tapestation (Agilent). Basecalling was performed using guppy with default parameters (https://nanoporetech.com/software/other/guppy; Table 1). Adapters were trimmed using *Q*-score trimming (Q30) and Porechop (v0.2.4; https://github.com/rrwick/Porechop), and all sequencing reads were further quality controlled and species confirmed (e.g., to exclude contamination) using Kraken 2 (8). Hybrid assembly using paired-end Illumina and long-read ONT reads was performed and circularized using Unicycler v0.4.8 (9), which additionally rotated the single contig to start with *dnaA*. The nullarbor pipeline (https://github.com/tseemann/nullarbor) was used for additional quality control and antimicrobial resistome (ResFinder v4.7.2) analysis. Default parameters were used for all software unless otherwise specified. The Prokaryotic Genome Annotation Pipeline v6.1 (10) provided by NCBI was used to identify and annotate the coding sequences (CDS) and RNAs in the genomes.

**TABLE 1:** Characteristics of *Haemophilus ducreyi* isolates ITG3105, CIP76118, and TMA3542; raw sequencing data; and complete reference genome features, including antimicrobial resistance determinants^*a*^

Features	ITG3105	CIP76118	TMA3542
Origin of the isolate	South Africa	France	Thailand
Year of isolation	1983	1978	1990
Source of the isolate	Chancroid	Chancroid	Chancroid
CCUG	14,233	7,470	27,022
Illumina short reads (*N*)	3,685,698	3,865,436	3,933,254
Average coverage depth	308×	326×	314×
Average Phred score	>Q30	>Q30	>Q30
Average read length (bp)	144	145	142
ONT long reads (*N*)	94,199	1,415,965	97,218
Average coverage depth	112×	4,626×	354×
Average Phred score	Q25	Q19	Q25
Average read length (bp)	5,126	4,684	3,100
Read N50	2,399	4,676	6,778
Maximum read length (bp)	116,716	243,471	102,388
Assembled sequences	3	1	4
Chromosome length (bp)	1,725,365	1,721,101	1,750,314
GC content (%)	38.3	38.3	38.0
CDS (*N*)	1,716	1,701	1,727
rRNA	19	19	19
tRNA	49	47	47
tmRNA	1	1	1
Plasmids	2	0	3
Antibiotic resistance determinants			
*aph*(3’)-Ia	–^*b*^	–	Yes
*aph*(3'')-Ib	–	–	Yes
*bla*TEM-1B	Yes	–	Yes
*bla*ROB-1	–	–	Yes
*catS*	–	–	Yes
*sul2*	Yes	–	Yes
*tet*(32)	–	Yes	Yes
*tet*(B)	Yes	–	Yes

^*a*^ *N*, number; ONT, Oxford Nanopore Technologies; bp, base pairs; CDS, coding sequence; CCUG, Culture Collection University of Gothenburg.

^*b*^ “–” indicates that the specific antimicrobial resistance determinants were not detected in the isolates.

Characteristics of the *H. ducreyi* isolates, sequencing data, and complete reference genomes (i.e., structural completeness: fully circularized, hybrid assemblies without gaps), including AMR determinants, are summarized in Table 1. Briefly, the ITG3105 genome consists of a single circular chromosome of 1,725,365 bp (encoding 1,716 CDS, 19 rRNAs, 49 tRNAs, and 1 tmRNA), and two plasmids: pDG001 (9,154 bp; 8 CDS) and pDG002 (7,584 bp; 9 CDS). The CIP76118 genome has a chromosome of 1,721,101 bp (1,701 CDS, 19 rRNAs, 47 tRNAs, and 1 tmRNA) and no plasmids. The TMA3542 genome has a chromosome of 1,750,314 bp (1,727 CDS, 19 rRNAs, 47 tRNAs, and 1 tmRNA) and three plasmids: pDG001 (9,154 bp; 8 CDS), pDG003 (5,685 bp; 6 CDS), and pDG004 (4,625 bp; 5 CDS). These complete reference genomes (chromosomes and plasmids) provide valuable resources for comparative genomics and molecular epidemiological surveillance of *H. ducreyi*, including monitoring of AMR determinants.

## Data Availability

The *H. ducreyi* ITG3105 (CCUG 14233), CIP76118 (CCUG 7470), and TMA3542 (CCUG 27022) isolates are available at Culture Collection University of Gothenburg (CCUG; https://www.ccug.se). ITG3105 originated from a human chancroid infection contracted in South Africa in 1983, CIP76118 from a chancroid case in France in 1978, and TMA3542 from a chancroid case in Bangkok, Thailand, in 1990. The genome assemblies and raw reads have been deposited under the following accession numbers: SAMN53162900 for ITG3105 chromosome, and plasmids pDG001 and pDG002; SAMN53162901 for CIP76118 chromosome; SAMN53162902 for TMA3542 chromosome and plasmids pDG001, pDG003, and pDG004. Short Read Archive: Illumina reads, ITG3105 (SRR35993037), CIP76118 (SRR35993039), and TMA3542 (SRR35993041); Nanopore reads, ITG3105 (SRR35993036), CIP76118 (SRR35993038), and TMA3542 (SRR35993040). The project summary is found under BioProject accession number PRJNA1359372.
